# Supplementary material for: miR449 Protects Airway Regeneration by Controlling AURKA/HDAC6-Mediated Ciliary Disassembly
Source: Int J Mol Sci. 2022 Jul 13;23(14):7749. doi: 10.3390/ijms23147749 (PMC9320302; doi:10.3390/ijms23147749)
Supplement: Supplementary file 1 [file ijms-23-07749-s001.zip › ijms-1764863-supplementary/ijms-1764863-supplementary.pdf]

Supplementary information

miR449 protects airway cilia and healthy lung aging.

Merit Wildung, Christian Herr, Dietmar Riedel, Cornelia Wiedwald, Alena Moiseenko, Fidel Ramírez, Hataitip Tasena, Maren Heimerl, Mihai Alevra, Naira Movsisyan, Maike Schuldt, Larisa Volceanov-Hahn, Sharen Provoost, Tabea Nöthe-Menchen, Diana Urrego, Bernard Freytag, Julia Wallmeier, Christoph Beisswenger, Robert Bals, Maarten van den Berge, Wim Timens, Pieter S. Hiemstra, Corry-Anke Brandsma, Tania Maes, Stefan Andreas, Irene H. Heijink, Luis A. Pardo and Muriel Lizé*

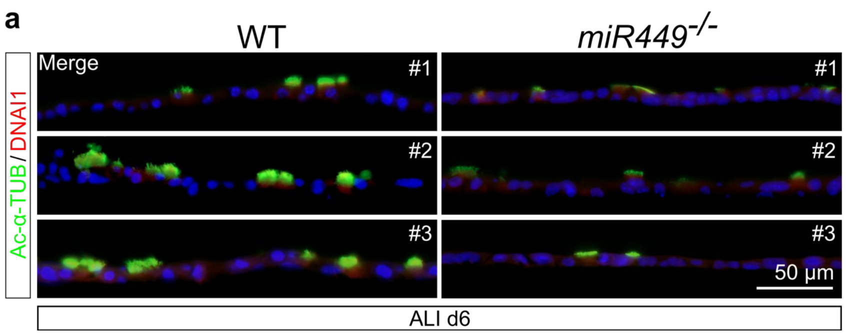

Supplementary Figure S1: *miR449*$^{-/-}$ ALI cultures express less cilia markers than their WT counterparts. a) Immunofluorescence staining of axonemal cilia markers Ac-α-TUB (green) and DNAI1 (red) in WT and *miR449*$^{-/-}$ ALI cultures at day (d) 6. Nuclei were counterstained with DAPI (blue). Images from *n*=3 ALI cultures per genotype. For one ALI culture 3 mice were used.

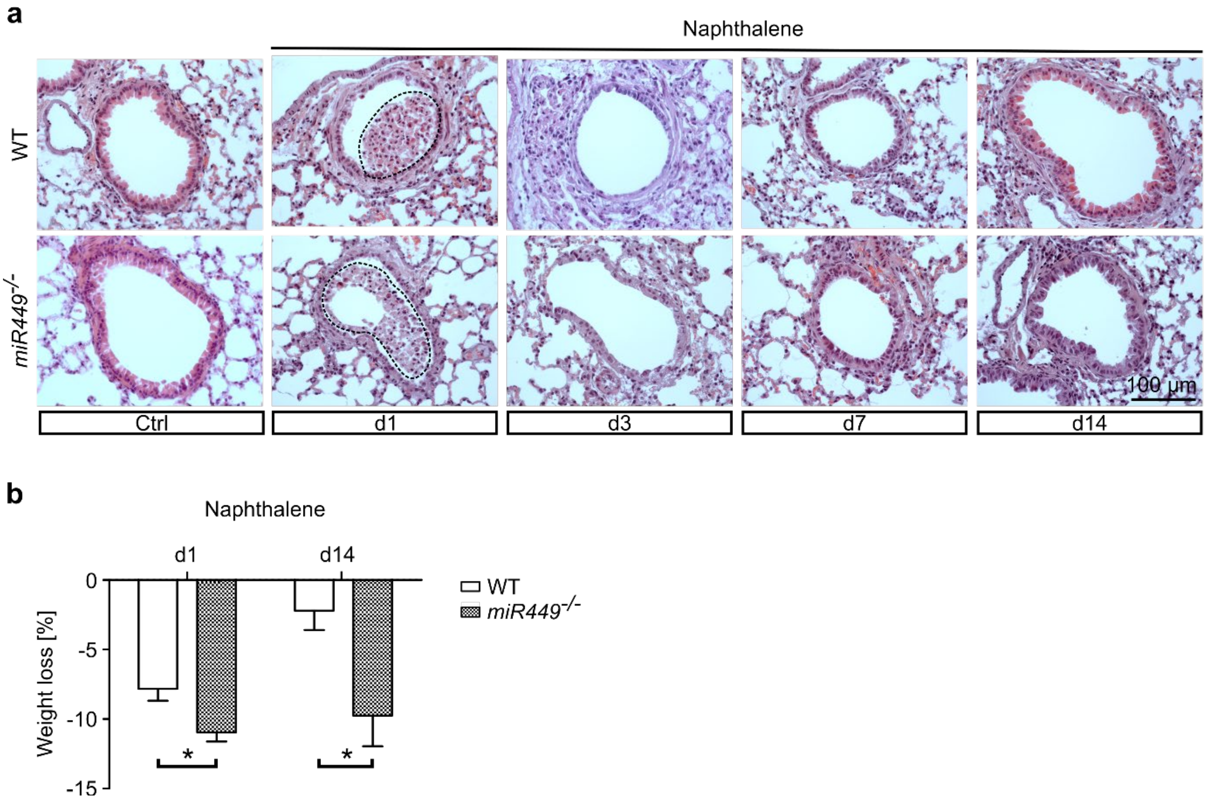

Supplementary Figure S2: Naphthalene treatment affects lung histology in WT and $miR449^{-/-}$ mice to a similar extent. a) Hematoxylin and eosin staining (H&E) staining of lung sections from oil (Ctrl) and naphthalene treated WT and $miR449^{-/-}$ mice. Control mice (oil) show a pseudostratified bronchial epithelium. After naphthalene injection at d1, injured Club cells exfoliate and accumulate in the airway lumen. Exfoliated Club cells are marked by a dotted circle at d1. **b)** Weight of WT and $miR449^{-/-}$ mice was recorded before naphthalene injection (d0) and at different time points after injection (d1, d14). Weight loss [%] represents the difference between the weight before and after naphthalene injection. WT: n=10 (d1) and n=5 (d14) mice per group; $miR449^{-/-}$: n=9 (d1), and n=4 (d14) mice per group. Data are presented as the mean ± SEM with $p < 0.05$.

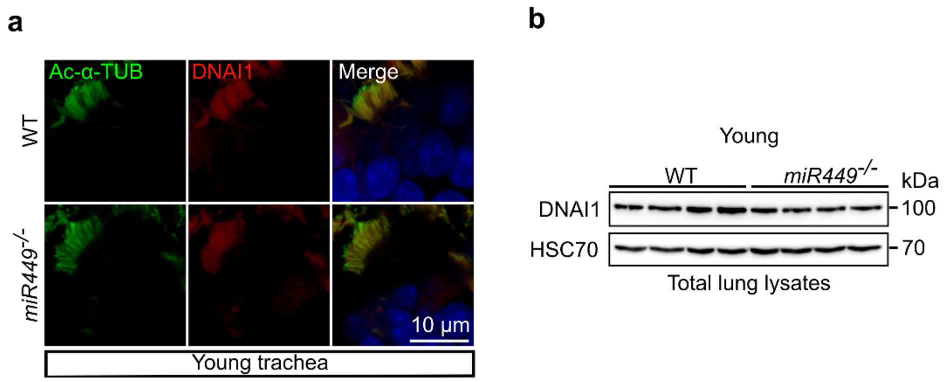

Supplementary Figure S3: Ciliation appears normal in young *miR449*$^{-/-}$ mice. a) Cryosections of 10 weeks-old WT and *miR449*$^{-/-}$ trachea were stained with Ac-α-TUB (green) and DNAI1 (red). Nuclei were counterstained with DAPI (blue). Images were taken with a confocal microscope. **b)** Lung lysates from 10-weeks-old WT and *miR449*$^{-/-}$ were separated by SDS-PAGE and analyzed by immunoblot using DNAI1 as a motile cilia marker and the constitutive protein HSC70 as a loading control.

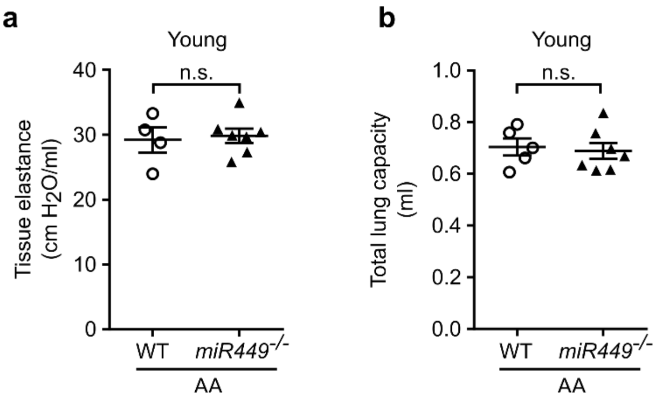

Supplementary Figure S4: Young *miR449*$^{-/-}$ mice do not display signs of spontaneous COPD. Pulmonary function was assessed in 12 weeks old WT and *miR449*$^{-/-}$ mice by measuring the tissue elastance **(a)** and total lung capacity **(b)** using the FlexiVent system. *n*=4-7 mice/genotype. Data are presented as the mean ± SEM. n.s=non-significant.

a

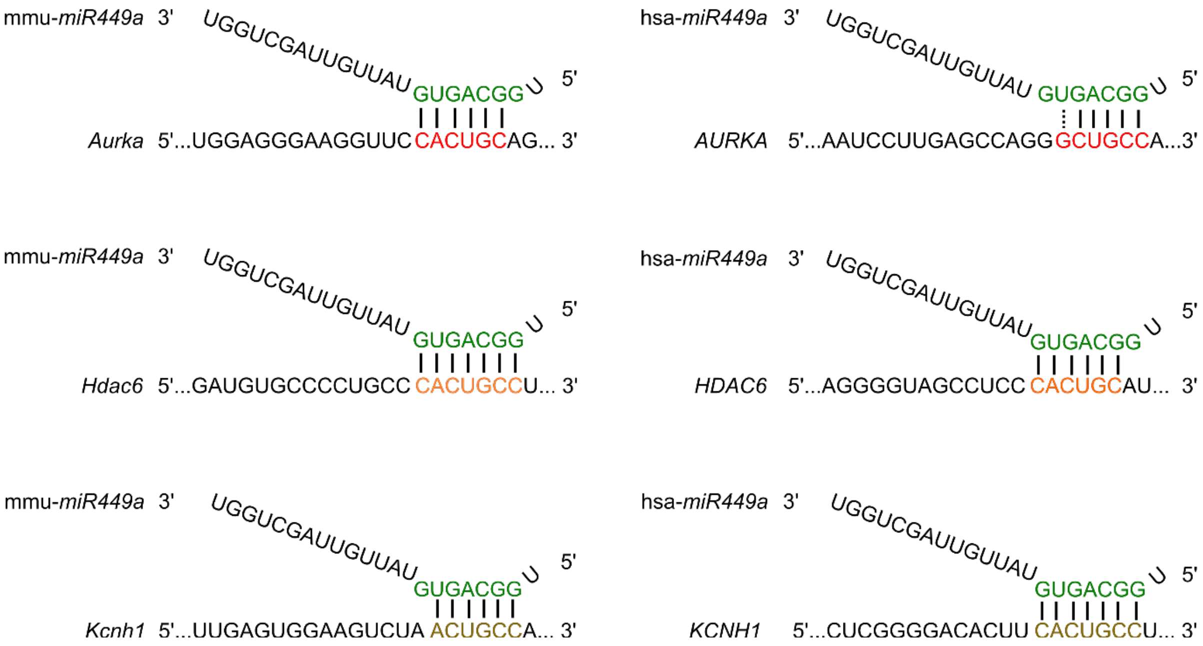

Supplementary Figure S5: Predicted targets of *miR449*. Mouse and human AURKA[1], HDCA6[2] as well as KCNH1[3] are predicted targets of *miR449*. The seed sequence of *miR449a* is shown in green.

References

1. Otto, T. *et al.* Cell cycle-targeting microRNAs promote differentiation by enforcing cell-cycle exit. *Proceedings of the National Academy of Sciences of the United States of America* **114,** 10660–10665 (2017).
2. Song, R. *et al.* miR-34/449 miRNAs are required for motile ciliogenesis by repressing cp110. *Nature* **510,** 115–120 (2014).
3. Lin, H. *et al.* Transcriptional and Post-Transcriptional Mechanisms for Oncogenic Overexpression of Ether À Go-Go K+ Channel. *PloS one* **6** (2011).

Supplementary Tables

Supplementary Table S1: Characteristics of COPD patients enrolled in the GLUCOLD study.

Characteristic	COPD patients (n = 57)
Gender (male), n (%)	48 (84.2)
Ex-smokers, n (%)	35 (61.4)
Pack-year*	41.5 (31.9-53.3)
Age, year*	59 (54-66)
FEV_1, %predicted*	64.3 (58.5-69.8)
FEV_1/FVC*	0.50 (0.44-0.56)

*Data are presented as median (interquartile range); FEV1 is forced expiratory volume in 1 second; FVC is forced vital capacity

Supplementary Table S2: Top 10 enriched biological processes among *miR34a-5p*-positively correlated genes in COPD patients.

NAME	NOM p-val	FDR q-val
DNA_DAMAGE_RESPONSE_SIGNAL_TRANSDUCTION_RESULTING_IN_TRANSCRIPTION	<0.001	1,0000
EPOXYGENASE_P450_PATHWAY	0,0039	0,9255
AXONEMAL_DYNEIN_COMPLEX_ASSEMBLY*	0,0094	0,8557
MICROTUBULE_BUNDLE_FORMATION*	0,0018	0,6606
GLYCERALDEHYDE_3_PHOSPHATE_METABOLIC_PROCESS	0,0020	0,5371
KERATINIZATION	0,0036	0,4565
AXONEME_ASSEMBLY*	<0.001	0,3979
GLUCOSE_6_PHOSPHATE_METABOLIC_PROCESS	0,0057	0,4535
HISTONE_H3_DEACETYLATION	0,0054	0,4680
MORPHOGENESIS_OF_A_POLARIZED_EPITHELIUM	0,0018	0,4294

*Cilia-associated processes are highlighted in green; GSEA was performed using the list of genes ranked based on the strength of their correlation with *miR34a-5p* and the Gene Ontology dataset for biological processes.

Supplementary Table S3: Top 10 enriched biological processes among *miR34b-5p*-positively correlated genes in COPD patients.

NAME	NOM p-val	FDR q-val
CILIUM_MORPHOGENESIS*	<0.001	<0.001
CILIUM_ORGANIZATION*	<0.001	<0.001
CELLULAR_COMPONENT_ASSEMBLY_INVOLVED_IN_MORPHOGENESIS	<0.001	<0.001
CELL_PROJECTION_ASSEMBLY	<0.001	<0.001
AXONEME_ASSEMBLY*	<0.001	<0.001
CILIUM_MOVEMENT*	<0.001	<0.001
MICROTUBULE_BUNDLE_FORMATION*	<0.001	<0.001
MICROTUBULE_BASED_MOVEMENT*	<0.001	<0.001
ORGANELLE_ASSEMBLY	<0.001	<0.001
PROTEIN_TRANSPORT_ALONG_MICROTUBULE*	<0.001	<0.001

*Cilia-associated processes are highlighted in green; GSEA was performed using the list of genes ranked based on the strength of their correlation with *miR34b-5p* and the Gene Ontology dataset for biological processes.

Supplementary Table S4: Primary antibodies.

Antibodies	Dilution (Application)	Company	Catalog [clone]
**Mouse monoclonal anti-Ac-α-TUB**	1:1,000 (IF-P)<br>1:4,000 (IF-Fr)	Merck	T6793 [6-11B-1]
**mouse monoclonal anti-Ac-α-TUB**	1:250 (ICC)	Santa Cruz (*Dallas, TX, USA*)	sc23950
**Rabbit polyclonal anti-β-ACTIN**	1:10,000 (WB)	Abcam	ab8227
**mouse monoclonal anti-Aurora A**	1:700 (WB)	Santa Cruz	sc56881
**Goat polyclonal Anti-CD206**	1:750 (WB)	*Novus Biological (Littleton, USA)*	AF2535
**Rabbit polyclonal anti-DNAI1**	1:500 (IF-P), 1:300 (IF-Fr), 1:700 (WB)	Merck	HPA021649
**Mouse monoclonal anti-HSC70**	1:20,000 (WB)	Santa Cruz	sc-7298 [B-6]
**Rabbit polyclonal anti-Pericentrin**	1:1,500 (ICC)	Abcam	ab4448
**Mouse monoclonal anti-TIMP1**	1:500	Invitrogen, Thermo Fisher Scientific	MA1-773

ICC = Immunocytochemistry, IF = Immunofluorescence, Fr = Frozen cryosections, P = Paraffin embedded sections, WB = Western blot.

Supplementary Table S5: Secondary antibodies.

Antibodies	Dilution (Application)	Company	Catalog #
**Alexa Fluor 488 donkey anti-mouse**	1:500 (IF)	Invitrogen, Thermo Fisher Scientific	A21202
**Alexa Fluor 488 goat anti-mouse**	1:1,000 (ICC)	Invitrogen, Thermo Fisher Scientific	A-11001
**Alexa Fluor 594 goat anti-rabbit**	1:500 (IF)	Invitrogen, Thermo Fisher Scientific	A11012
**Peroxidase-conjugated donkey anti-mouse**	1:10,000 (WB)	Jackson Immunoresearch	715-036-150
**Peroxidase-conjugated donkey anti-goat**	1:10,000 (WB)	Jackson Immunoresearch	705-036-147
**Peroxidase-conjugated donkey anti-rabbit**	1:10,000 (WB)	Jackson Immunoresearch (*West Grove, PA, USA*)	711-036-152

ICC = Immunocytochemistry, IF = Immunofluorescence, WB= Western blot

Supplementary Table S6: Sequence information for primers used in RT-qPCR.

Gene	Forward primer (5` to 3`)	Reverse primer (5` to 3`)
hsaAURKA	TTCTTCCCAGCGCATTCCTT	TTCCTTTACCCAGAGGGCGA
mmuCdc20b	CTCGCCAACGGCATGAAGCAG	CTCCGCAGACAGCCTCTTCACG
mmuDnah5	CTGACGGACGCTGGGGACAC	CACTGGGGTGGTCGCCGAAG
mmuMmp9	CTGGACAGCCAGACACTAAAG	TCTCGCGGCAAGTCTTCAGAG
mmuMmp12	GGGCTGCTCCCATGAATGAC	CCAGAGTTGAGTTGTCCAGTTG
